# Patient-reported outcome measures in drugs for neurological conditions approved by European Medicines Agency 2017–2022

**DOI:** 10.1007/s10072-023-06825-6

**Published:** 2023-05-05

**Authors:** Oriana Ciani, Michela Meregaglia, Mario Alberto Battaglia, Gianpaolo Brichetto, Antonella Conte, Claudio Gasperini, Valeria Sansone

**Affiliations:** 1 Government, Health & Not-for-Profit Division, Center for Research On Health and Social Care Management, SDA Bocconi School of Management, Health Economics & HTA, MEO Building, Room W210, II Floor, Via Sarfatti 10, 20136 Milan, Italy; 2 Italian Multiple Sclerosis (AISM) Society, Genoa, Italy; 3 Associazione Italiana Sclerosi Multipla (AISM) Rehabilitation Center, Genoa, Italy; 4 Department of Human Neurosciences, Sapienza, University of Rome, Rome, Italy; 5 IRCCS Neuromed, Pozzilli, Italy; 6 Italian Society of Neurology (SIN), Siena, Italy; 7 Department of Neurosciences, S. Camillo Forlanini Hospital, Rome, Italy; 8 NeMO Clinical Center, Neurorehabilitation Unit, University of Milan, Milan, Italy

**Keywords:** Patient-reported outcomes, European Medicines Agency

## Abstract

**Background:**

Regulatory agencies have been responsive to public demand for inclusion of the patient experience in evaluating and approving therapies. Over the years, patient-reported outcome measures (PROMs) have become increasingly prevalent in clinical trial protocols; however, their influence on regulators, payers, clinicians, and patients’ decision-making is not always clear. We recently conducted a cross-sectional study aimed at investigating the use of PROMs in new regulatory approvals of drugs for neurological conditions between 2017 and 2022 in Europe.

**Methods:**

We reviewed European Public Assessment Reports (EPARs) and recorded on a predefined data extraction form whether they considered PROMs, their characteristics (e.g., primary/secondary endpoint, generic/specific instrument) and other relevant information (e.g., therapeutic area, generic/biosimilar, orphan status). Results were tabulated and summarized by means of descriptive statistics.

**Results:**

Of the 500 EPARs related to authorized medicines between January 2017 and December 2022, 42 (8%) concerned neurological indications. Among the EPARs of these products, 24 (57%) reported any use of PROMs, typically considered as secondary (38%) endpoints. In total, 100 PROMs were identified, of which the most common were the EQ-5D (9%), the SF-36 (6%), or its shorter adaptation SF-12, the PedsQL (4%).

**Conclusions:**

Compared to other disease areas, neurology is one where the use of patient-reported outcomes evidence is inherently part of the clinical evaluation and for which core outcome sets exist. Better harmonization of the instruments recommended for use would facilitate the consideration of PROMs at all stages in the drug development process.

## Introduction

Recently, there has been a growing interest in how to incorporate a patient-centered approach in the regulatory evaluation of drugs’ efficacy and safety profile. Among different potential options, patient-reported outcome measures (PROMs) are standardized, validated instruments that are completed directly by patients to capture their perspective of their health condition, physical, social, emotional functioning, quality of life, and well-being [1].

Regulatory agencies have been responsive to public demand for inclusion of the patient experience in evaluating and approving therapies. Over the years, PROMs have become increasingly more prevalent in clinical trial protocols [2], although their influence on regulators, payers, clinicians, and patients’ decision-making is not always clear [3].

Among the different public health authorities responsible for the evaluation and licensing of pharmaceutical products, the European Medicines Agency (EMA) has championed the adoption of a patient-centered approach to clinical research [4]. In particular, the agency’s strategic view to 2025 [5] reinforces the need to systematically incorporate the patient voice throughout drug development and evidence generation. We recently conducted a study aimed at investigating the use of PROMs in new regulatory approvals of drugs for diseases of the nervous system between 2017 and 2022 in Europe by reviewing their corresponding European Public Assessment Reports (EPARs) [6].

## Methods

Among the EPARs for drugs authorized between 2017 and 2022, we identified those related to the diseases of the nervous system using the ICD-10 classification (G00-G99). The data extraction was performed by two reviewers independently, with a third involved to solve disagreements. An ad hoc template was created in Microsoft® Excel to systematically record the use of PROs and/or PROMs for each EPAR and other relevant information (i.e., drug’s characteristics including trade name, active substance, authorization/refusal year, generic/biosimilar, orphan status). For each specific PROM retrieved, we looked for the associated underlying PRO concept to identify the PRO-PROM dyads; if both PRO and PROM were missing, we concluded that the EPAR did not show any patient-reported evidence. Therefore, we categorized the type of PRO endpoint (i.e. primary, secondary, or other) included in registrative trials and the type of corresponding PROM (i.e., generic or specific). The extracted data were analyzed through descriptive statistics to investigate the use of PROs/PROMs over time and drug’s characteristics associated with this use. Moreover, for drugs reporting at least one PRO/PROM dyad, we identified the corresponding clinical trials on clinicaltrials.gov (using the study code reported in the EPAR) to confirm the use of PRO/PROM in the underlying clinical trial protocol.

## Results

**Fig. 1:**
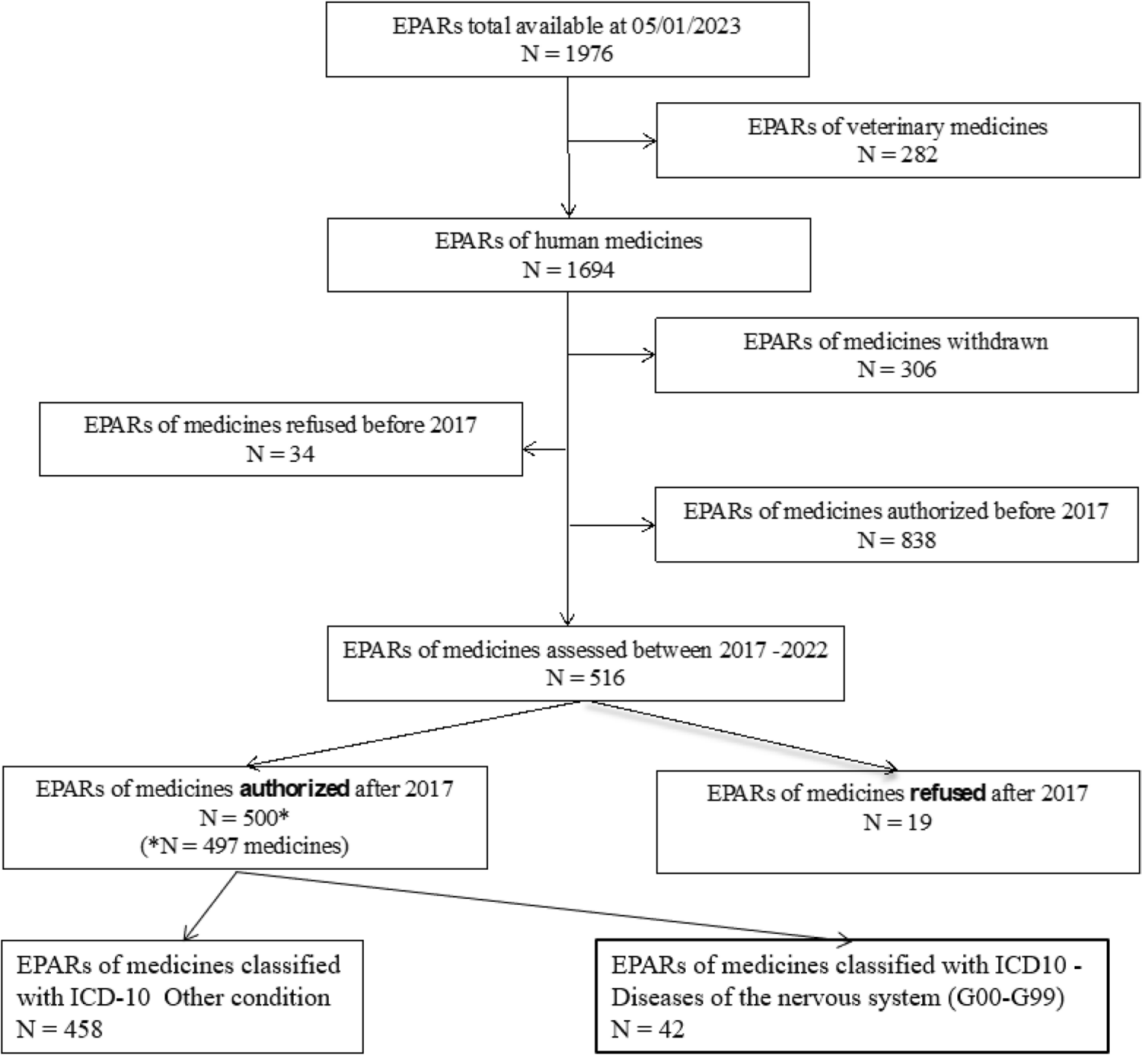
Flow chart of EPARs selection

Of the 1976 drugs identified on the EMA website, we excluded drugs for veterinary use (*n* = 282) and drugs withdrawn (*n* = 306), refused (*n* = 53), or authorized before 2017 (*n* = 838). Therefore, we obtained a final list of 500 EPARs related to 497 authorized medicines between January 2017 and December 2022, of which 42 (8.4%) concerned neurological indications (Fig. 1).

**Fig. 2:**
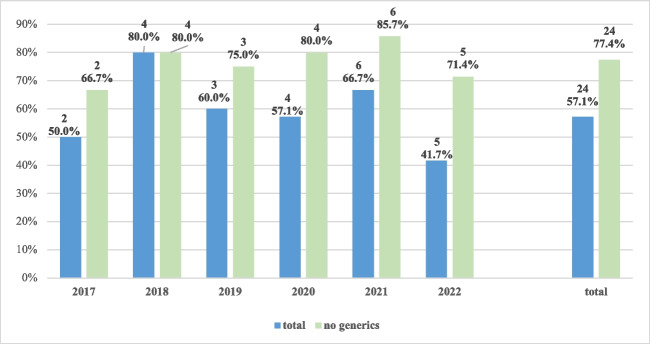
Use of PROs/PROMs in EMA drug approvals for nervous system diseases (2017–2022)

The indications covered were multiple sclerosis (36%), epilepsy (14%), migraine (14%), spinal muscular atrophy (7%), sleep disorders (11.9%), or other conditions (17%). Among the EPARs of these products, 24 out of 42 (57%) reported any use of PROs/PROMs. The protocols of clinical trials supporting the authorization reported about PRO/PROM endpoint in 96% of cases (23 out of 24 drugs); however, this does not guarantee that results on those outcomes have been reported in clinical studies. When excluding generic drugs (*n* = 11), consideration of such measures increased from 57% to 77% (24 out of 31) and also slightly improved over time (from 67% in 2017 to 71% in 2022) (Fig. 2). On top of non-generic status, the frequency of use of patient-reported evidence was far more common for drugs with orphan designation (6 out of 8, 75%).

The PRO represented the primary endpoint of the clinical efficacy evaluation in six cases (25%), but more often was considered as secondary endpoint (38%); health-related quality of life (HRQoL) was the underlying construct to be assessed in most cases (30%).

In total, 100 PROMs were identified, of which the most common were EQ-5D (9%), SF-36, or its shorter adaptation SF-12 (6%), PedsQL (4%), and PGI-C (4%), all well-validated generic HRQoL instruments for adults or children/young people. As disease-specific measures, the EPARs mainly reported Migraine Disability Assessment (MIDAS, 5%), Quality of Life in Childhood Epilepsy Questionnaire (QOLCE, 5%), e-diary for headache (4%), Migraine-Specific Quality of Life Questionnaire (MSQ, 3%), Epworth Sleepiness Scale (ESS, 3%), Headache Impact Test (HIT-6, 3%), and Insomnia Daytime Symptoms and Impacts Questionnaire (IDSIQ, 3%). Of the 100 PROMs, 15 (15%) were proxy-reported by caregivers or specifically intended for caregivers. Among the 24 EPARs reporting any use of PROs/PROMs, the mean number of measures was 4.1, and the median was 3 (range: 1–11).

## Discussion

Compared to other disease areas, neurology is one where the use of PROs evidence is inherently part of the clinical evaluation and for which researchers and principal investigators internationally have developed over time more than 16 different recommended core outcome sets to be measured in clinical trials which includes at least a PROM [7]. Other areas showing similar awareness are rheumatology, orthopedics and trauma, lungs and airways, and dermatology. Most frequently used tools are generic questionnaires for the assessment of HRQoL, such as SF-36 or EQ-5D; however, there is a plethora of additional disease-specific instruments developed and used in practice which target typically physical, social, cognitive, and emotional functional domains. Interestingly, 15 (15%) of the PROMs in this sample were proxy-reported, indicating the importance to capture subjective feedback on functioning and symptoms in kids or individuals with cognitive problems. Proxy-reported measures are deemed most appropriate to capture children’s outcomes since they are not considered to have the skills needed to express complex concepts such as thoughts and feelings and to answer HRQoL questionnaires, and PROMs in general, reliably. Obtaining this type of information is now possible thanks to innovations in modern measurement theory, qualitative methods for instrument development, and computerized technologies to create reliable and valid methods for obtaining self- and proxy-reported health data among the young population [8]. The variety of PROMs recorded in this evaluation of EPARs does not fit with a vision of systematic, harmonized collection of PROM data even within each disease indication and reveals lack of a much-needed effort to agree on standardized measurement tools within patient populations and across key target domains. Better harmonization of the instruments recommended for use would facilitate the consideration of PROMs at all stages in the drug development process. This harmonization would also benefit the implementation of PROMs monitoring in clinical practice. Despite claims that it can at time be time-consuming, it is usually well accepted by patients, and current technological advancement allows for continuous and remote (at home or in the waiting room) completion by the patient and/or caregiver, meaning that the healthcare professionals can focus on the interpretation of the replies given and their concordance with other objective measures [9]. The return is better knowledge about the disease burden and understanding of treatments’ impact on health-related quality of life and subjectively reported symptoms, which can complement the clinical assessment and often inform the choice of treatment of disease-related insidious symptoms (e.g., pain, fatigue).

The strategic vision launched by EMA in 2020 aims to reinforce patient relevance in evidence generation for pharmaceutical products through a coordinated approach to patient-reported outcomes collection and promotion of core HRQoL instruments. This study however does not show an increase in PROs/PROMs consideration before and after 2020 across the whole sample of drug authorizations, but only among the non-generic pharmaceutical products. In the fall 2022, the Agency published an executive summary stemming from a multi-stakeholder workshop on patient experience data in medicines development and regulatory decision-making [10]. The document recognizes that patients’ perspectives on medicines and their benefits and risks are of great value to EMA and the EU Regulatory Network, as patients can provide valuable insights and perspectives from living with a condition and its treatment, as well as information about outcomes and preferences that are important for future treatments. Our findings suggest that approximately two out of three drug approvals in neurology take into consideration information directly collected from patients about symptoms or physical functioning in pivotal trials. Efficient PRO data collection would provide a significant incentive to an increased uptake of such measures not only in clinical research, but also in real-world settings. Digital health solutions for monitoring electronic PRO (e-PRO) now allow for a wide-scale, standardized, continuous collection of PROMs, once issues of interoperability, data governance, security, privacy, logistics, and ethics have been addressed [11]. Selection of appropriately validated and agreed upon PROs represents a major opportunity to account for what matter most for patients affected by neurological conditions and ultimately improve their health outcomes.

## Data Availability

Data are available upon reasonable request to the authors.
